# Healthcare seeking behaviour among self-help group households in Rural Bihar and Uttar Pradesh, India

**DOI:** 10.1186/s12913-015-1254-9

**Published:** 2016-01-04

**Authors:** Wameq A. Raza, Ellen Van de Poel, Pradeep Panda, David Dror, Arjun Bedi

**Affiliations:** 1 Institute of Health Policy and Management, Erasmus University Rotterdam, J5-23, P.O. Box 1738, 3000 DR Rotterdam, The Netherlands; 2 Research and Evaluation Division, BRAC, Dhaka, Bangladesh; 3 Institute of Health Policy and Management, Erasmus University Rotterdam, Rotterdam, The Netherlands; 4 Micro Insurance Academy, New Delhi, India; 5 Micro Insurance Academy, India and Erasmus University Rotterdam, The Hague, The Netherlands; 6 International Institute of Social Studies (ISS), Erasmus University Rotterdam, The Netherlands and Georgetown University, Doha, Qatar

**Keywords:** Healthcare seeking behaviour, Non-degree allopathic providers, Community-based health insurance, Self-help group, India

## Abstract

**Background:**

In recent years, supported by non-governmental organizations (NGOs), a number of community-based health insurance (CBHI) schemes have been operating in rural India. Such schemes design their benefit packages according to local priorities. This paper examines healthcare seeking behaviour among self-help group households with a view to understanding the implications for the benefit packages offered by such schemes.

**Methods:**

We use cross-sectional data collected from two of India’s poorest states and estimate an alternative-specific conditional logit model to examine healthcare seeking behaviour.

**Results:**

We find that the majority of respondents do access some form of care and that there is overwhelming use of private providers. Non-degree allopathic providers (NDAP) also called rural medical practitioners are the most popular providers. In the case of acute illnesses, proximity plays an important role in determining provider choice. For chronic illnesses, cost of care influences provider choice.

**Conclusion:**

Given the importance of proximity in determining provider choice, benefit packages offered by CBHI schemes should consider coverage of transportation costs and reimbursement of foregone earnings.

**Electronic supplementary material:**

The online version of this article (doi:10.1186/s12913-015-1254-9) contains supplementary material, which is available to authorized users.

## Background

Healthcare financing in India is still largely reliant on out of pocket spending (OOPS),^1^ exposing many households to financial hardship or causing them to forego care altogether [1–3]. Since 2008, the government has been offering inpatient coverage through a scheme called Rashtriya Swasthya Bima Yojana (RSBY) for those below the poverty line, but outpatient care, representing some 80 % of total health expenditure, is generally still not included [4, 5]. In the absence of other solutions to ease OOPS, a number of non-governmental organizations (NGOs) have introduced community-based health insurance (CBHI) schemes in rural India [6, 7]. These schemes have different benefit-packages, reflecting both different priorities within a demand-driven model, and unequal availability of services across rural locations.^2^ Clearly, a good understanding of household healthcare seeking behaviour can inform how well such schemes respond to perceived priorities.

There is some evidence on determinants of health-seeking behaviour in urban settings in India [8–14]. However, studies based on rural India are comparatively sparse [4, 15–17]. Ager and Pepper [18] reported that in 1996 primary healthcare centres were relatively underused in rural Odisha and that households preferred (qualified and unqualified) private providers.^3^ They reported that reputation of provider, cost and ease of access were important in influencing provider choice. Using data from India’s National Sample Survey Organisation (NSSO), Borah [19] and Sarma [20] found that the demand for healthcare in rural areas is negatively affected by the price of healthcare and distance to a healthcare facility. They concluded that poorer households were more price-sensitive, with higher elasticity of demand in seeking care for children than for adults. Gautham, et al. [21], using data from household surveys, key informant interviews and focus group discussions, found that the majority (92 %) of respondents in Andhra Pradesh visited private providers, of which 75 % visited non-degree allopathic providers (NDAP); and in Odisha, 53 % of respondents sought allopathic care, of which about 76 % were NDAP. The main reasons for such choices were providers’ proximity, and their readiness to make home visits when needed.

The main objective of this paper is to examine and understand healthcare seeking behaviour with a view to drawing lessons on the design of benefit packages offered through CBHI schemes. In particular, this paper provides evidence on the healthcare seeking behaviour of a specific but important group in rural India, namely households affiliated to self-help groups (SHG). The study was carried out against the backdrop of the introduction of CBHI schemes, implemented by local NGOs, which were going to offer insurance to households where at least one member was affiliated to a self-help group in March 2010 (see Doyle et al., [22] for further details). The study draws on baseline surveys which were conducted a year prior to scheme launch and focuses on rural Uttar Pradesh and Bihar, two of India’s most populated, poorest and least urbanized states, with large gender differences. 4 As SHG households are typically poorer and less educated than the general population our analysis sheds light on the healthcare seeking behaviour of a relatively marginalized population in rural India [23].^5^

We begin the analysis by estimating the probability of seeking any out or inpatient care. Second, we model the probability of seeking care from a specific provider, while distinguishing between patient and provider characteristics. Third, our analysis distinguishes between care sought for acute and chronic conditions, between outpatient and inpatient care, and we examine the probability of seeking care from a wider range of providers.

The paper is organized as follows: the methods (data and analytical techniques) are described in section 2, followed by results in section 3. Section 4 contains a discussion and concluding remarks.

## Methods

### Data and specification

The data used in this paper is drawn from household surveys conducted between March and May 2010 in *Kanpur Dehat* and *Pratapgarh* districts in Uttar Pradesh and in *Vaishali* district in Bihar.^6^ As mentioned above, these baseline surveys preceded the implementation of three CBHI schemes which offered insurance to targeted households.^7^ The target group consisted of 3686 SHG households (1284 in Pratapgarh, 1039 in Kanpur Dehat and 1363 in Vaishali) representing 21,366 individuals. All targeted households were surveyed. The primary respondents were the SHG members themselves or the head of the household, if the member was unavailable. Information on other household members was collected from the primary respondents.^8^

While the survey gathered information on a wide range of socio-demographic and economic characteristics, of particular interest is the detailed information collected on health status, self-reported symptoms experienced during the four weeks preceding the survey for outpatient care and one year for inpatient care, and choice of provider. Respondents who reported an illness were asked whether they sought care, and if so, from which type of provider. Data pertaining to the following pre-selected providers were collected: traditional healers, priests, pharmacists, NDAPs, nurses, qualified private doctors, qualified public doctors, specialist public doctors, specialist private doctors and ‘others’.^9^

Outpatient episodes were separated into acute or chronic.^10^ For chronic illnesses, information was gathered on the most recent visit; for acute illnesses, information was gathered for up to three illnesses and three visits per illness in the four weeks preceding the survey. While we have data on multiple illnesses and multiple visits, the analysis deals mainly with choice of healthcare provider for the first illness and the first visit, as most individuals (98 %) experienced only a single illness during the four-week period. While there are repeat-visits for the same illness, the number of cases is not as large as the first visit and perhaps more importantly, as will be discussed later, the choice of provider does not vary substantially in subsequent visits. In the case of inpatient care the survey enquired whether any household member had been hospitalized in the 12 months preceding the survey.

Consistent with the existing literature, the probability of healthcare use and the choice of provider are modelled as functions of individual and household level covariates [19, 20, 24]. The individual characteristics include the respondent’s demographics, educational attainment, occupational status and self-reported health status. For models related to acute illnesses, we use the socioeconomic characteristics of the household head, since a substantial proportion of the sample consists of children (41 %). We control for the nature of the respondent’s illness by including a set of self-reported symptom variables and health status is measured by the generic quality of life variable (EQ5D) which contains information on five dimensions of health: mobility, self-care, pain, ability to perform usual activities and mental health status. The scores from each question are converted into an index that is increasing in health and ranges between −1 to +1 using the procedure suggested by Dolan [25]. As these questions were administered only to individuals older than 12 years, the EQ5D measure is only used while modelling the probability of obtaining care for chronic conditions which is estimated only for respondents older than 12. Household level covariates include household size and gender of the household head, whether a household belongs to a scheduled tribe or caste and household socioeconomic status as captured by (the log of) per capita consumption.^11^

### Analytical technique

The probabilities of using acute and chronic outpatient care, and inpatient care, are modelled using probit specifications. We consider the probability of using outpatient care conditional on reporting an illness while for the probability of inpatient care we use the full sample.

To model the choice of healthcare provider for outpatient care, we use an alternative-specific conditional logit model [26]. This has the advantage of allowing both individual and provider level characteristics to influence the choice of healthcare provider [19, 27, 28] and does not require arbitrary choices as in the case of a nested logit model [29–31]. The probability that individual *i* chooses healthcare provider *j* (out of a set of *m* providers) can then be written as:

1 $$ {\mathrm{p}}_{\mathrm{i}\mathrm{j}}=\frac{ \exp \left({\mathrm{x}}_{\mathrm{i}\mathrm{j}}^{\hbox{'}}\upbeta +{\mathrm{z}}_{\mathrm{i}}^{\hbox{'}}{\upgamma}_{\mathrm{j}}\right)}{{\displaystyle {\sum}_{\mathrm{l}=1}^{\mathrm{m}}} \exp \left({\mathrm{x}}_{\mathrm{i}\mathrm{l}}^{\hbox{'}}\upbeta +{\mathrm{z}}_{\mathrm{i}}^{\hbox{'}}{\upgamma}_{\mathrm{l}}\right)},\ \mathrm{j}=1,\dots,\ \mathrm{m} $$

where ${x}_{ij}$ are healthcare provider specific regressors and ${z}_i$ are individual specific regressors.

Since respondents only report information such as cost and travel time for the providers they actually visit, following established practice [19, 27, 32], we impute costs and time faced by each individual and for each provider.^12^ We estimate a log linear model on the sample of users (using individual, household covariates and village indicator variables) and subsequently predict costs and travel time for the entire sample.^13^ To ease interpretation of coefficients, we calculate marginal effects for the alternative specific variables as:

2 $$ \frac{\delta {p}_{ij}}{\delta {x}_{ik}}={p}_{ij}\left(1-{p}_{ij}\right)\beta $$

Since we use the logarithm of costs and travel time in our models, the marginal effects for these two variables is interpreted as the change in the probability of choosing healthcare provider *j* due to a 1 % increase in costs or travel time. All analysis was done using STATA version 12.0.

## Results

### Summary statistics

Table 1 shows summary statistics for the full sample, and separately for the three different sites. Half of the adult respondents are women while children younger than 13 years account for 37 % of the sample. The average household size is 6.8. About 37 % of household heads have no education while 11 % have higher secondary education. As for employment, 34 % of the household heads are self-employed in agriculture followed by 26 % who work as casual wage labourers. Thirty percent of the sample may be classified as scheduled caste or tribe (SC/ST).^14^ The average annual per capita consumption is INR 13,588.^15^ While there are differences across the three sites in aspects such as the percentage of female headed households and occupational status of household head, differences are minimal for household size, self-assessed health status, educational attainment, share of SC/ST, and annual per capita expenditures.

**Table 1:** Description and means of covariates

Variable	Means			
	Pooled	Kanpur Dehat	Pratapgarh	Vaishali
Demographics				
Female headed household (1/0)	0.19	0.09	0.19	0.21
Female children 0–13 (1/0)	0.18	0.15	0.16	0.20
Female aged 14–55 years (1/0)	0.29	0.27	0.31	0.27
Female older than 55 years (1/0)	0.04	0.06	0.04	0.05
Male aged 0–13 years (1/0)	0.19	0.16	0.19	0.21
Male aged 14–55 years (1/0)	0.26	0.31	0.26	0.23
Male older than 55 years (1/0)	0.04	0.05	0.04	0.04
Household size	6.77 (2.75)	6.94 (2.64)	7.28 (3.22)	6.10(2.07)
Self-assessed health measure (EQ5D) increasing in health (−1 to +1)	0.76	0.77	0.79	0.72
Education (respondent)				
No education (1/0)	0.38	0.33	0.35	0.44
Primary education (1/0)	0.26	0.24	0.26	0.28
Secondary education (1/0)	0.28	0.33	0.30	0.23
Higher secondary education (1/0)	0.08	0.10	0.09	0.05
Education of household head				
No education (1/0)	0.37	0.31	0.33	0.46
Primary education (1/0)	0.17	0.14	0.19	0.17
Secondary education (1/0)	0.35	0.40	0.37	0.28
Higher secondary education (1/0)	0.11	0.15	0.11	0.09
Socioeconomic Status				
Annual per capita expenditure (Indian Rupees [INR])	13588 (17329)	15922 (25338)	11368 (10095)	13961 (14688)
Household belongs to a scheduled tribe/caste (1/0)	0.30	0.28	0.33	0.29
Occupation (respondent)				
Self-employed in agriculture (1/0)	0.11	0.19	0.07	0.07
Self-employed in non-agriculture (1/0)	0.04	0.03	0.05	0.06
Other employment (1/0)	0.02	0.02	0.04	0.02
Casual wage labourer (1/0)	0.09	0.05	0.09	0.11
Not working (1/0)	0.06	0.05	0.07	0.04
Doing housework (1/0)	0.20	0.21	0.20	0.19
Student (1/0)	0.48	0.45	0.48	0.51
Occupation of household head				
Self-employed in agriculture (1/0)	0.34	0.63	0.22	0.21
Self-employed in non-agriculture (1/0)	0.14	0.07	0.17	0.18
Other employment (1/0)	0.07	0.03	0.12	0.05
Casual wage laborer (1/0)	0.26	0.13	0.29	0.35
Not working (1/0)	0.07	0.05	0.11	0.04
Doing housework (1/0)	0.12	0.09	0.09	0.17
Student (1/0)	0.00	0.00	0.00	0.00
Location				
Household located in Kanpur Dehat (1/0)	0.29			
Household located in Pratapgarh (1/0)	0.37			
Household located in Vaishali (1/0)	0.34			

Underlined categories are used as reference categories in the regression models. The health status indicator EQ5D only pertains to those above the age of 12. Standard deviation provided in parentheses for continuous variables. *N* = 21,366

### Disease burden and healthcare seeking behaviour

**Fig. 1:**
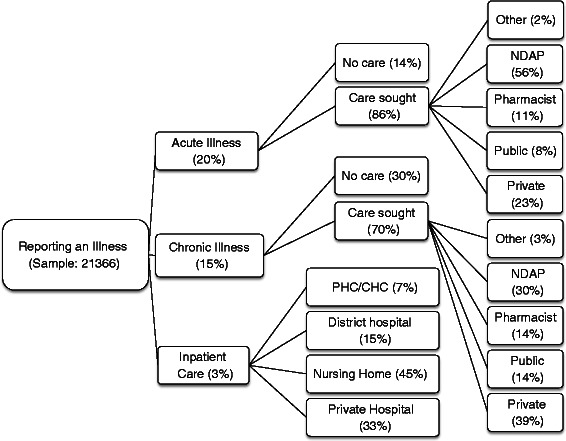
Health seeking behaviour in the sample. Legend: The sample for chronic illnesses and inpatient care exclude children younger than 13 years of age

Table 2 shows the distribution of self-reported symptoms for both acute and chronic conditions. Approximately 20 % and 15 % of individuals report acute and chronic symptoms, respectively (see Fig. 1).^16^ Over half (52 %) of the acute conditions relate to gastrointestinal symptoms (diarrhoea and cholera), followed by respiratory symptoms (20 %). While symptoms related to chronic conditions were more difficult to classify, 27 % were grouped into the ‘other’ category, followed by musculoskeletal symptoms (23 %), lung/respiratory symptoms (15 %) and gastrointestinal symptoms (15 %). Ten percent of the sample reports having persistent allergies or infections.

**Table 2:** Distribution of self-reported symptoms for acute and chronic conditions

	Category	Means			
		Pooled	Kanpur Dehat	Pratapgarh	Vaishali
Acute (*N* = 4171)	Gastrointestinal symptoms (1/0)	0.52	0.57	0.56	0.46
	Febrile symptoms (1/0)	0.08	0.06	0.05	0.14
	Lungs/respiratory symptoms (1/0)	0.20	0.15	0.21	0.22
	Musculoskeletal symptoms (1/0)	0.04	0.03	0.05	0.03
	Other symptoms (1/0)	0.16	0.19	0.13	0.15
Chronic (*N* = 3277)	Lungs/respiratory symptoms (1/0)	0.15	0.17	0.09	0.21
	Gastrointestinal symptoms (1/0)	0.15	0.13	0.14	0.19
	Musculoskeletal symptoms (1/0)	0.24	0.20	0.35	0.09
	Chronic allergies/infections (1/0)	0.10	0.08	0.09	0.14
	Other symptoms (1/0)	0.27	0.33	0.24	0.28
	Internal symptoms (1/0)	0.09	0.09	0.10	0.09

Underlined categories are used as reference categories in the regression models. Chronic conditions exclude children younger than 13 years of age

**Fig. 2:**
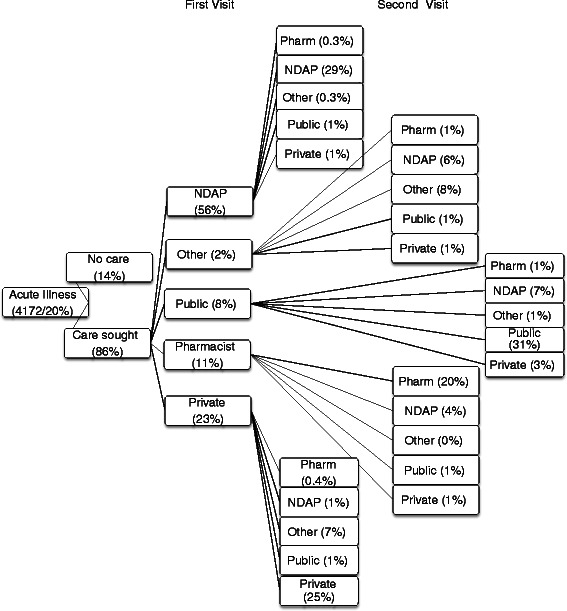
Health seeking behaviour for those suffering from an acute illness (first and second visit). Legend: Proportions of the second visit do not cumulate to 100 %. The difference represent individuals who do not seek care the second time

Figure 1 displays the pattern of healthcare seeking behaviour in the sample (see Additional file 1: Table S1 for site level details). There are several notable points emerging from the figure. The majority of individuals do seek care for both acute (86 %) and chronic illnesses (70 %). Of those who seek care for acute illnesses, only 8 % visit qualified doctors/specialists at public health facilities while the rest seek care from private practitioners. NDAPs account for 56 % of visits while qualified doctors/specialists in private practice account for 23 % of the visits, followed by pharmacists (11 %). For chronic illnesses the private sector dominates (83 % of healthcare visits). Qualified private doctors/specialists and NDAPs are responsible for a substantial proportion of care (39 % and 30 % respectively) followed by pharmacists (14 %). With regard to inpatient care, once again private care (nursing homes and private hospitals) dominates and accounts for 78 % of visits followed by public hospitals (15 %) and other public providers (7 %). Figure 2 displays healthcare seeking behaviour for second visits in the case of acute illnesses. The main point emerging from the figure is that individuals tend to use the same provider a second time. For instance of the 1991 individuals who visited NDAPs, 629 (29 %) report a second visit of which 91 % visit an NDAP. In the case of those who visited private providers, 35 % report a second visit of which 72 % visit a private provider the second time around.

### Determinants of seeking care conditional upon reporting illness

Table 3 contains estimates of the probability of seeking outpatient care for acute (column 1), chronic illnesses (column 2), and inpatient care (column 3). Several points emerge from these probit estimates. Across all three specifications, for the most part, employment status and whether an individual belongs to the SC/ST groups do not have much of a bearing on the probability of seeking care. However, socioeconomic status as reflected by annual per capita household expenditure is positively correlated with the probability of seeking care. A one percent increase in expenditure is associated with a four percentage point (pp) increase in the probability of seeking care in case of an acute illness. The effect for chronic illnesses is stronger (seven percentage point effect) while for inpatient care the effect is much smaller, perhaps reflecting the necessity of such care. Reflecting ease of access to at least some form of medical care, educational attainment is not correlated with the probability of seeking care for acute illnesses. However, those with higher educational levels (higher secondary education) are substantially (13 pp) more likely to seek care for chronic illnesses. In the case of acute illnesses there are clear gender differences. Male children (0–13 years) and working age men (14–55 years) are more likely to be treated for acute conditions compared to adult females (5 and 6 percentage points respectively). Female children are also more likely to receive care compared to adult females in the age group 14 to 55. Respondents in Pratapgarh and Kanpur Dehat are substantially less likely to seek outpatient care compared to those in Vaishali. This may be due to the greater proximity of healthcare providers in Vaishali versus the other two sites. The health status of an individual has an expected sign, namely those in better health are less likely to seek care.

**Table 3:** Determinants of the probability of seeking outpatient care for acute and chronic conditions and of seeking inpatient care

Variable	Acute Illness (1)		Chronic Illness (2)		Inpatient Care (3)	
	Marginal Effects	Standard Error	Marginal Effects	Standard Error	Marginal Effects	Standard Error
Female headed household (1/0)	0.002	0.018	−0.033^a^	0.018	−0.000	0.003
Female children 0–13 (1/0)	0.035^b^	0.015				
Female older than 55 years (1/0)	0.012	0.026	0.016	0.024	−0.011^b^	0.005
Male aged 0–13 years (1/0)	0.050^c^	0.015				
Male aged 14–55 years (1/0)	0.060^c^	0.015	−0.017	0.021	−0.006^a^	0.003
Male older than 55 years (1/0)	0.042	0.033	0.012	0.026	−0.010^b^	0.005
Log of household size	0.058^c^	0.017	0.107^c^	0.020	−0.003	0.003
Primary education (1/0)	0.017	0.017	0.026	0.021	0.007^a^	0.003
Secondary education (1/0)	−0.016	0.013	−0.003	0.019	0.005^a^	0.003
Higher secondary education (1/0)	0.022	0.020	0.131^c^	0.031	0.004	0.004
Natural log of annual per capita expenditure (INR)	0.037^c^	0.013	0.069^c^	0.019	0.005^a^	0.003
Scheduled caste/tribe (1/0)	−0.004	0.012	−0.008	0.015	−0.001	0.003
Self-employed in non-agriculture (1/0)	0.002	0.018	0.041	0.032	−0.005	0.005
Other employment (1/0)	−0.066^c^	0.022	0.080^b^	0.038	0.005	0.006
Casual wage labourer (1/0)	−0.017	0.015	0.003	0.026	0.003	0.004
Not working (1/0)	−0.033	0.022	−0.015	0.027	0.000	0.005
Doing housework (1/0)	−0.011	0.022	0.001	0.022	0.000	0.004
Student (1/0)			−0.033	0.031		
Kanpur Dehat	−0.098^c^	0.015	−0.087^c^	0.019	−0.006^b^	0.003
Pratapgarh	−0.029^b^	0.014	−0.101^c^	0.017	−0.007^c^	0.003
Acute gastrointestinal symptoms (1/0)	−0.032^b^	0.015				
Acute febrile symptoms (1/0)	−0.093^c^	0.022				
Acute musculoskeletal symptoms (1/0)	−0.136^c^	0.026				
Other acute symptoms (1/0)	−0.020	0.019				
Self-assessed health measure Increasing in health (−1 to +1)			−0.150^c^	0.024	−0.049^c^	0.004
Chronic lungs/respiratory symptoms (1/0)			0.043^a^	0.023		
Chronic gastrointestinal symptoms (1/0) (1/0)			0.061^c^	0.023		
Chronic allergic symptoms (1/0)			−0.004	0.026		
Other chronic symptoms (1/0)			0.008	0.019		
Chronic symptoms related to Internal organs (1/0)			0.021	0.027		
*N*	4,171		3,276		13,965	

The table provides marginal effects based on probit models. Models for outpatient care are estimated over the sample of respondents that reported an illness. The sample for chronic illnesses and inpatient care exclude children younger than 13 years of age. The employment and occupation variables refer to the employment and occupation of the household head. ^a^,^b^,^c^ indicate significance at the 10,5 and 1 % respectively

### Determinants of choice of provider

**Fig. 3:**
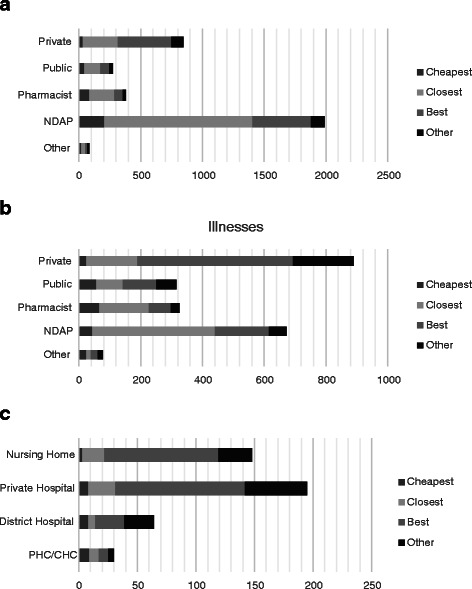
**a** Reasons for choosing provider for acute illnesses. **b** Reasons for choosing provider for chronic illnesses. **c** Reasons for choosing provider for inpatient care. Self-reported reasons for choosing a healthcare provider for acute, chronic and inpatient conditions. Legend: Each figure for acute, chronic and inpatient care represents the number of cases (3573, 2280 and 437 respectively) reported. The sample for chronic illnesses and inpatient care exclude children younger than 13 years of age. Responses are not mutually exclusive

Figure 3 shows the main reasons provided by respondents for choosing a specific healthcare provider (Fig. 3a for acute, Fig. 3b for chronic conditions and Fig. 3c for inpatient care respectively). In the case of acute illnesses, NDAPs dominate and the main reason for visiting them is their proximity (60 %), followed by the view that they are the best providers (23 %) while cost considerations are not as important (10 %). Those who visit private hospitals point out that the main reason for visiting them is that they are considered the best providers of care (50 %) followed by proximity. With regard to chronic conditions, qualified doctors/specialists in private practice dominate as they are considered as best by the care-seekers (58 %). The reason for visiting NDAPs is their proximity. Disaggregated results by site reveal similar patterns (see Additional file 2: Table S2).

Before modelling health provider choice, we estimated travel time and average costs for providers across sites, both for acute and chronic conditions (Additional file 3: Table S3). Across all three sites the closest providers are NDAPs followed by pharmacists (17 and 19 min travel time, respectively). On average, qualified public and private providers are about 40 min away. Across the three locations Vaishali seems to have the greatest concentration of access to healthcare facilities. On average, NDAPs are only 9 min away in Vaishali as compared to 18 and 24 min in Pratapgarh and Kanpur Dehat respectively. Similarly, it takes about 32 min to access qualified doctors in Vaishali as compared to 54–56 min in Kanpur Dehat.

With regard to the costs of treatment, there are marked differences across acute and chronic conditions. Regardless of the provider, the cost of care is higher for treating chronic conditions compared to acute illnesses. We find that pharmacists are the cheapest amongst the various providers for both acute and chronic illnesses (INR 69 and INR 154 respectively), followed by NDAPs (INR 128 and INR 246 respectively), public doctors (INR 155 and INR 570 respectively) and private doctors (INR 380 and INR 929 respectively).

Table 4 shows odds ratios (OR) based on a conditional logit model for choice of outpatient care for acute conditions (reference category: public healthcare providers). Children, either male or female, are more likely to receive care from private doctors or NDAPs. There is some evidence that higher education is associated with the use of greater care from private providers. For instance, households headed by heads that have secondary education are more likely to use private care (OR – 1.7) and individuals living in households where heads have higher secondary education are less likely to visit NDAPs (OR – 0.65). Patients living in households with higher per capita annual expenditure are less likely to forego care and also less likely to visit other providers. Belonging to a SC/ST group has no bearing on provider choice. Consistent with the differences in availability of care, respondents living in Kanpur Dehat are more likely to forego care while households in Pratapgarh are far more likely to use public care as compared to other providers.

**Table 4:** Determinants of provider choice for outpatient care for acute conditions

Variable	None		Other		Pharmacist		Private		NDAP	
	Odds Ratio	Standard Error	Odds Ratio	Standard Error	Odds Ratio	Standard Error	Odds Ratio	Standard Error	Odds Ratio	Standard Error
Female headed household (1/0)	1.336	0.348	3.054^c^	1.233	1.215	0.339	1.414	0.356	1.435	0.335
Female children 0–13 (1/0)	1.382	0.413	2.016	1.123	1.32	0.417	2.308^c^	0.672	1.861^b^	0.487
Female older than 55 years (1/0)	1.104	0.437	1.427	0.877	1.311	0.565	1.346	0.522	1.215	0.439
Male aged 0–13 years (1/0)	1.087	0.31	2.875^b^	1.535	0.865	0.264	2.200^c^	0.605	1.673^b^	0.412
Male aged 14–55 years (1/0)	0.742	0.158	1.191	0.472	1.321	0.295	1.411^a^	0.283	1.175	0.218
Male older than 55 years (1/0)	0.78	0.358	2.794^a^	1.697	1.009	0.509	1.064	0.467	1.152	0.465
Log of household size	0.540^c^	0.124	0.555	0.232	0.614^a^	0.153	0.938	0.201	0.954	0.19
Primary education (1/0)	0.859	0.195	0.292^b^	0.142	0.841	0.204	1.064	0.228	1.11	0.216
Secondary education (1/0)	1.517^b^	0.295	1.005	0.351	1.149	0.242	1.694^c^	0.317	1.264	0.219
Higher secondary education (1/0)	0.707	0.187	0.912	0.502	0.937	0.259	1.371	0.332	0.654^a^	0.149
Log of annual per capita exp. (INR)	0.650^b^	0.117	0.462^b^	0.164	0.78	0.155	0.963	0.156	0.909	0.14
Scheduled caste/tribe (1/0)	1.129	0.188	0.659	0.2	1.042	0.188	0.91	0.147	1.209	0.178
Self-employed in non-agriculture (1/0)	1.19	0.303	1.139	0.479	1.867^b^	0.483	1.540^a^	0.363	0.967	0.212
Other employment (1/0)	1.662^a^	0.467	0.819	0.443	1.473	0.441	1.115	0.308	0.738	0.186
Casual wage labourer (1/0)	1.369	0.296	1.231	0.483	1.155	0.271	1.486^a^	0.305	1.128	0.213
Not working (1/0)	1.953^b^	0.635	0.825	0.646	1.071	0.404	2.115^b^	0.666	1.375	0.398
Doing housework (1/0)	0.945	0.305	0.605	0.318	0.841	0.297	1.157	0.358	0.784	0.225
Student (1/0)	0.752	0.183	0.476	0.226	1.007	0.257	0.606^b^	0.143	0.818	0.172
Kanpur Dehat	1.992^c^	0.458	1.181	0.413	0.420^c^	0.11	0.653^a^	0.143	1.261	0.263
Pratapgarh	0.435^c^	0.087	0.203^c^	0.072	0.408^c^	0.082	0.193^c^	0.036	0.389^c^	0.068
Acute gastrointestinal symptoms (1/0)	1.446^a^	0.296	1.621	0.602	1.265	0.267	0.834	0.156	1.201	0.209
Acute febrile symptoms (1/0)	1.718^a^	0.533	1.216	0.751	0.701	0.246	0.736	0.221	0.78	0.219
Acute musculoskeletal symptoms (1/0)	4.711^c^	2.112	4.894^b^	3.199	2.121	1.026	1.801	0.814	1.343	0.58
Other acute symptoms (1/0)	0.901	0.223	1.212	0.515	0.645	0.173	0.97	0.216	0.643^b^	0.135
Log of cost	0.996	0.005	0.996	0.005	0.996	0.005	0.996	0.005	0.996	0.005
Log of time	0.858^c^	0.029	0.858^c^	0.029	0.858^c^	0.029	0.858^c^	0.029	0.858^c^	0.029
*N = 4171*										

The table provides odds ratios based on a conditional logit model. The reference category is visiting a public provider. Models are estimated over the sample that reported an acute illness. The employment and occupation variables refer to the employment and occupation of the household head. ^a^, ^b^, ^c^ indicate significance at the 10, 5 and 1 % respectively

The last two rows of Table 4 illustrate that respondents are sensitive to the time it takes to reach a provider, and are far less likely to visit providers located further away. Table 5 shows the marginal effects of travel time required to reach various types of providers. A 1 % increase in travel time reduces the probability of visiting a NDAP by 4%age points and the probability of visiting a private doctor by 2 percentage points. Respondents are not as responsive in the case of travel time to pharmacists and public doctors. Consistent with Fig. 3, these estimates show that the main advantage of NDAP is their proximity. The substantially larger negative effect of distance to NDAPs compared to more qualified providers suggests that if NDAPs were not located close by, their advantage would be whittled away as households would then be less likely to trade proximity for quality. Surprisingly, and an issue that we return to later, the cost of care does not seem to have a bearing on provider choice.

**Table 5:** Predicted probabilities of the effect of travel time to the provider

	Travel Time		Cost	
	Acute Illness	Chronic Illness	Acute Illness	Chronic Illness
No care	−0.018^c^	−0.020	0.000	−0.021^c^
Other	−0.003^c^	−0.002	−0.001	−0.003^c^
Pharmacy	−0.012^c^	−0.008	0.000	−0.009^c^
Private	−0.024^c^	−0.018	−0.001	−0.019^c^
Public	−0.009^c^	−0.008	−0.001	−0.009^c^
NDAP	−0.036^c^	−0.015	0.000	−0.017^c^

^c^indicate significance at the 1 % respectively

Estimates pertaining to chronic illnesses are provided in Table 6. There is no strong statistical evidence of gender related differences. If anything, it seems that older males are more likely to forego care (OR – 2.16). Households headed by individuals with higher secondary education are far more likely to visit private providers (OR – 3.4). Caste and household per capita expenditure do not seem to exert a strong influence on provider choice. In contrast to the findings for acute illnesses, we find that travel time does not influence provider-choices. However, provider choice is sensitive to cost (last two rows of Table 6). A one percent increase in cost reduces the probability of visiting an NDAP or a private doctor by 2 percentage points (Table 5).

**Table 6:** Determinants of provider choice for outpatient care for chronic conditions

Variable	None		Other		Pharmacist		Private		NDAP	
	Odds Ratio	Standard Error	Odds Ratio	Standard Error	Odds Ratio	Standard Error	Odds Ratio	Standard Error	Odds Ratio	Standard Error
Female headed household (1/0)	0.635	0.213	0.738^a^	0.125	0.814^a^	0.101	0.806	0.137	0.863	0.114
Female older than 55 years (1/0)	1.478	0.633	0.810	0.193	0.951	0.170	0.812	0.200	1.209	0.218
Male aged 14–55 years (1/0)	1.268	0.473	0.816	0.166	1.010	0.147	0.806	0.161	1.075	0.170
Male older than 55 years (1/0)	2.157^a^	0.929	0.893	0.225	1.006	0.192	0.844	0.220	1.164	0.232
Log of household size	1.708	0.560	1.455^a^	0.298	1.807^c^	0.264	1.502^b^	0.310	1.642^c^	0.258
Self-assessed health measure Increasing in health (−1 to +1)	1.307	0.592	0.439^c^	0.101	0.434^c^	0.075	0.328^c^	0.073	0.588^c^	0.110
Primary education (1/0)	0.963	0.372	1.163	0.232	1.164	0.176	1.394	0.292	1.065	0.170
Secondary education (1/0)	1.053	0.326	0.871	0.162	1.134	0.149	1.326	0.239	0.799	0.114
Higher secondary education (1/0)	0.655	0.396	1.909^b^	0.596	2.596^c^	0.582	3.360^c^	0.965	1.382	0.350
Log of annual per capita exp. (INR)	2.034^c^	0.558	1.174	0.228	1.560^c^	0.203	1.126	0.218	1.212	0.175
Scheduled caste/tribe (1/0)	1.306	0.343	0.996	0.140	0.848	0.090	0.968	0.139	1.144	0.125
Self-employed in non-agriculture (1/0)	1.766	0.896	0.868	0.257	1.308	0.287	0.848	0.268	1.396	0.333
Other employment (1/0)	2.847^a^	1.651	1.576	0.513	1.370	0.393	2.261^b^	0.753	1.999^b^	0.572
Casual wage labourer (1/0)	1.071	0.545	0.726	0.173	1.135	0.201	0.896	0.224	0.972	0.189
Not working (1/0)	1.203	0.543	0.646	0.173	1.152	0.225	0.807	0.221	1.051	0.217
Doing housework (1/0)	1.404	0.557	0.654^b^	0.136	1.043	0.165	0.994	0.213	1.124	0.189
Students	0.786	0.463	0.513^b^	0.164	0.870	0.191	0.889	0.259	0.920	0.221
Kanpur Dehat	1.358	0.460	0.247^c^	0.061	0.491^c^	0.068	0.841	0.168	1.159	0.179
Pratapgarh	0.695	0.233	0.844	0.141	0.346^c^	0.042	0.703^b^	0.126	0.754^b^	0.103
Chronic lungs/respiratory symptoms (1/0)	2.153^a^	0.909	0.608^b^	0.145	1.520^b^	0.255	0.979	0.231	1.217	0.205
Chronic gastrointestinal symptoms (1/0)	3.616^c^	1.450	1.326	0.266	1.725^c^	0.295	0.839	0.205	1.632^c^	0.272
Chronic allergic symptoms (1/0)	1.321	0.638	0.632^a^	0.157	1.185	0.219	1.021	0.244	0.701^a^	0.141
Other chronic symptoms (1/0)	1.924^a^	0.713	0.602^c^	0.110	1.616^c^	0.230	1.264	0.229	0.837	0.122
Chronic symptoms related to Internal organs (1/0)	2.304^a^	1.006	0.617^a^	0.163	1.647^c^	0.308	1.033	0.260	0.819	0.167
Log cost	0.841^c^	0.030	0.841^c^	0.030	0.841^c^	0.030	0.841^c^	0.030	0.841^c^	0.030
Log time	1.055	0.044	1.055	0.044	1.055	0.044	1.055	0.044	1.055	0.044
*N = 3276*										

The table provides odds ratios based on a conditional logit model. The reference category is visiting a public provider. Models are estimated over the sample that reported a chronic illness. Sample excludes children below 13 years of age. The employment and occupation variables refer to the employment and occupation of the household head. ^a^, ^b^, ^c^ indicate significance at the 10, 5 and 1 % respectively

## Discussion and conclusion

This paper examined healthcare seeking behaviour among households where at least one female member is affiliated to a woman’s self-help group in rural parts of Bihar and Uttar Pradesh, India. Consistent with recent evidence from rural Odisha, a state in Eastern India [21], we found that the majority of rural households do access some form of care. In the case of acute illnesses only 14 percent of respondents forego care and in the case of chronic illnesses about 30 % do not seek care.

Analysis of provider usage patterns shows overwhelming use of private care for both outpatient and inpatient services. In the case of acute illnesses, private care is sought by 90 % of those who seek care while the corresponding figures are 83 % in the case of chronic illnesses and 78 % in the case of hospitalization. This study confirms the findings of Ager and Pepper [18] and Gautham et al. [21] that non-degree allopathic providers account for a substantial proportion of health care. In this study such providers accounted for 56 % of all visits in acute cases and 30 % in the case of chronic illnesses (see Fig. 1). With regard to acute illnesses, the econometric estimates highlight the importance of proximity in determining provider choice while the self-reported information (Fig. 3) confirms that the main reason for relying so heavily on NDAP is their proximity. Somewhat different from findings reported in Borah [19] and Sarma [20], we found that direct costs did not have a bearing on choice of provider at least in the case of acute illnesses but does influence provider choice when households are faced with chronic illnesses. For chronic illnesses the econometric estimates show that cost plays a role in determining provider choice while proximity is not as important. This is consistent with the patterns in Fig. 1 which show that qualified private practitioners are the most sought after providers in case of chronic illnesses and that households rely on such providers as they are considered the best source of care (see Fig. 3). Overall, in the case of acute conditions, which are less likely to be serious, proximity appears to be important in driving provider choice while in the case of chronic conditions households feel the need for higher quality and costs are more likely to inhibit access to care. Notwithstanding these remarks, it is possible that identification of the cost effect is inhibited by the use of predicted cost variables, rather than actual information on costs of care across providers for different ailments.

Given the paper’s focus on households where women are affiliated to self-help groups, the generalizability of the findings may be limited. Furthermore, the lack of information on cost of care and other provider-specific factors such as quality of care are also limitations. Notwithstanding, these limitations our findings confirm that in the locations studied there is a tendency to seek care from allopathic providers, mostly unqualified, and that publicly provided services are less likely to be chosen, even by a relatively poor population in two of India’s poorest states.

This study has been conducted within the framework of a larger research program which deals with the implementation of CBHIs in rural India. A key implication from this study is that since proximity is an important factor influencing healthcare-seeking behaviour, CBHI schemes should also consider reimbursement for transportation costs and/or reimbursement of foregone earnings as part of the insurance package. Some experiments with CBHI in India and Nepal have already reported doing just that [7, 33]. Finally, one cannot ignore the preponderant role of NDAPs in provision of primary care. The debate over their role in the Indian rural medical provision system is well known [34, 35].

## Additional files

Additional file 1: Table S1. Pooled and site level pattern of healthcare seeking behavior in the sample areas. (DOCX 14 kb)

Additional file 2: Table S2. Self-reported reasons for choosing a healthcare provider for acute, chronic and inpatient conditions by site. (DOCX 15 kb)

Additional file 3: Table S3. Predicted means/standard deviations (SD) of estimated travel time and cost by provider. (DOCX 14 kb)

## Abbreviations

CBHI: Community based health insurance
NGOs: Non-government organizations
NDAP: Non-degree allopathic providers
RSBY: Rashtriya Swasthya Bima Yojana
OOPS: Out of pocket spending
NSSO: National sample survey organization
SHG: Self-help groups
GP: General practitioners
SC/ST: Scheduled tribes/Scheduled Castes

## Footnotes

1 Private expenditure constitutes 81 % of total health expenditure in India of which 94 % is out-of-pocket expenditure [4]. Less than 15 % of the population is covered by health insurance [4, 36].

2 There are a number of schemes operating across the country, each of which offers different benefit packages. Examples include the Yeshasvini CBHI programme in Karnataka which provides coverage for outpatient diagnosis, laboratory tests and inpatient surgical procedures, a scheme in Gudalur, Andhra Pradesh provides only hospitalization coverage [37, 38], and a scheme in Maharashtra is restricted to providing consultation services through community health workers [39]. Each of these schemes targets different groups in rural India - Yeshasvini targets farmers belonging to a co-operative and informal sector workers; the CBHI in Gudalur targets indigenous (Adivasi) households while the scheme in Maharashtra targets low-skilled workers.

3 Unqualified or less than fully qualified practitioners [34] are referred to by a variety of designations: rural medical practitioners (RMPs), local medical providers, non-degree allopathic providers or somewhat informally as “quacks”.

4 The surveys were designed to provide a better understanding of the healthcare needs among the targeted group such that the benefit package could be tailored to meet local needs.

5 A self-help group (SHG) usually consists of between 10–20 poor women living in the same village who come together and agree to save a specific amount each period. The savings of all SHG members are combined and deposited in a bank or a co-operative organization. Members may borrow from the pooled savings when the SHG agrees to give the loans. SHGs are usually supported and trained by NGOs. According to Fouillet, Augsburg [40] there are about 40 million SHG members in India.

6 These districts were purposively selected as the NGOs offering the CBHI insurance scheme had an established network of self-help groups in these three districts.

7 Project details can be found at http://www.microinsuranceacademy.org/project/uttar-pradesh-and-bihar-india/ and in Doyle, Panda et al. [22].

8 For all survey instruments, ethical approval was acquired from the independent ethics committee of the University of Cologne. Informed consent was taken prior to each interview, and respondents were free to halt the interview at any time, or to refuse to answer questions.

9 Qualified private doctors and specialists have been grouped together and are henceforth referred to as private doctors. Similarly, qualified public doctors and specialists have been grouped together and are referred to as public doctors. Due to the small number of observations, traditional healers, priests and nurses have been grouped together and form part of the ‘other’ category. We have also clubbed trained AYUSH (Ayurveda, Yoga and Naturopathy, Unani, Siddha and Homoeopathy) doctors in category “other” as only 2 of 4184 respondents who reported an illness sought care from an AYUSH doctor.

10 Chronic illnesses are defined as conditions that are reported to have been ongoing for 30 days or more.

11 Information on household consumption is self-reported and based on a 30-day recall period for store bought and home grown food items and a 12 month recall period for household durables and investments in agricultural equipment. This is then divided by the household size to arrive at the figure.

12 This study considers direct costs of care that relate to consultation fees, medicines, lab and imaging tests.

13 While we follow accepted practice, it is likely that this approach underestimates variation in costs and travel time that may be experienced by those who did not use care.

14 This is a higher proportion than the state rural SC/ST averages (nearly 17 % in Bihar and 23 % in Uttar Pradesh).

15 PPP$1 = INR 18.073 for 2010. A comparison between SHG affiliated households and randomly selected non-SHG affiliated households in the same location shows that the monthly per capita expenditure and educational attainment of SHG members was about 6 % and 7 % lower than the comparison group.

16 The surveys were implemented during the low morbidity season and it is possible that the incidence of reported illnesses is understated. In this part of rural India, the maximum burden of disease occurs during the monsoon season – May to July (see Kumari et al., [41]).
