# Supplementary material for: Healthcare seeking behaviour among self-help group households in Rural Bihar and Uttar Pradesh, India
Source: BMC Health Serv Res. 2016 Jan 4;16:1. doi: 10.1186/s12913-015-1254-9 (PMC4698810; doi:10.1186/s12913-015-1254-9)
Supplement: Supplementary file 1 — Pooled and site level pattern of healthcare seeking behavior in the sample areas. (DOCX 14 kb) [file 12913_2015_1254_MOESM1_ESM.docx]

## Supplementary Table 1: Pooled and site level pattern of healthcare seeking behavior in the sample areas

| **Description** | **Pooled** | **Kanpur Dehat** | **Pratapgarh** | **Vaishali** |
| --- | --- | --- | --- | --- |
| Probability of seeking care for acute illnesses (past month) (1/0) | 0.86 | 0.81 | 0.87 | 0.90 |
| Probability of seeking care for chronic illnesses (past month) (1/0) | 0.70 | 0.70 | 0.65 | 0.76 |
|  |  |  |  |  |
| *Type of health worker seen for acute illnesses* |  |  |  |  |
| None | 0.14 | 0.19 | 0.13 | 0.10 |
| Other | 0.02 | 0.02 | 0.01 | 0.03 |
| NDAP | 0.48 | 0.52 | 0.47 | 0.44 |
| Pharmacist | 0.09 | 0.04 | 0.13 | 0.11 |
| Public | 0.07 | 0.05 | 0.10 | 0.04 |
| Private | 0.20 | 0.18 | 0.16 | 0.28 |
|  |  |  |  |  |
| *Type of health worker seen for chronic illnesses* |  |  |  |  |
| None | 0.30 | 0.30 | 0.34 | 0.24 |
| Other | 0.02 | 0.03 | 0.03 | 0.02 |
| NDAP | 0.21 | 0.25 | 0.20 | 0.17 |
| Pharmacist | 0.10 | 0.04 | 0.13 | 0.09 |
| Public | 0.10 | 0.10 | 0.10 | 0.08 |
| Private | 0.27 | 0.28 | 0.20 | 0.40 |
|  |  |  |  |  |
| *Type of health facility visited for inpatient care* |  |  |  |  |
| PHC/CHC | 0.07 | 0.12 | 0.04 | 0.05 |
| District Hospital | 0.15 | 0.19 | 0.21 | 0.07 |
| Private Hospital | 0.44 | 0.52 | 0.40 | 0.41 |
| Nursing Home | 0.34 | 0.17 | 0.35 | 0.47 |

Notes: The sample for chronic illnesses and inpatient care exclude children younger than 13 years of age.
