# Supplementary material for: Healthcare seeking behaviour among self-help group households in Rural Bihar and Uttar Pradesh, India
Source: BMC Health Serv Res. 2016 Jan 4;16:1. doi: 10.1186/s12913-015-1254-9 (PMC4698810; doi:10.1186/s12913-015-1254-9)
Supplement: Supplementary file 2 — Self-reported reasons for choosing a healthcare provider for acute, chronic and inpatient conditions by site. (DOCX 15 kb) [file 12913_2015_1254_MOESM2_ESM.docx]

## Supplementary Table 2: Self-reported reasons for choosing a healthcare provider for acute, chronic and inpatient conditions by site

|  | **Kanpur Dehat** | | | | **Pratapgarh** | | | | **Vaishali** | | | |
| --- | --- | --- | --- | --- | --- | --- | --- | --- | --- | --- | --- | --- |
|  | Cheapest | Closest | Best | Other | Cheapest | Closest | Best | Other | Cheapest | Closest | Best | Other |
|  | **Acute Illnesses** | | | | | | | | | | | |
| Private | 7 | 90 | 115 | 26 | 7 | 78 | 129 | 8 | 17 | 118 | 186 | 67 |
| Public | 11 | 30 | 17 | 6 | 22 | 90 | 34 | 9 | 9 | 13 | 20 | 14 |
| Pharma | 19 | 28 | 7 | 2 | 34 | 112 | 23 | 9 | 30 | 66 | 38 | 13 |
| NDAP | 103 | 403 | 168 | 34 | 30 | 463 | 160 | 11 | 74 | 331 | 148 | 62 |
| Other | 8 | 18 | 2 | 2 | 5 | 6 | 4 | 1 | 5 | 9 | 12 | 10 |
|  | **Chronic Illnesses** | | | | | | | | | | | |
| Private | 7 | 47 | 119 | 37 | 10 | 65 | 210 | 25 | 7 | 54 | 174 | 134 |
| Public | 16 | 19 | 28 | 17 | 28 | 66 | 53 | 15 | 12 | 1 | 28 | 33 |
| Pharma | 10 | 10 | 6 | 2 | 42 | 121 | 40 | 10 | 14 | 29 | 26 | 15 |
| NDAP | 15 | 112 | 51 | 13 | 19 | 203 | 87 | 12 | 9 | 83 | 37 | 31 |
| Other | 5 | 6 | 6 | 5 | 13 | 8 | 10 | 7 | 5 | 1 | 7 | 5 |
|  | **Inpatient Care** | | | | | | | | | | | |
| PHC/CHC | 3 | 4 | 7 | 3 | 2 | 3 | 0 | 0 | 4 | 1 | 1 | 2 |
| District Hospital | 2 | 4 | 6 | 13 | 3 | 1 | 17 | 7 | 3 | 1 | 2 | 5 |
| Private Hospital | 3 | 7 | 45 | 20 | 4 | 5 | 30 | 13 | 1 | 11 | 36 | 20 |
| Nursing Home | 0 | 4 | 10 | 10 | 1 | 5 | 33 | 6 | 2 | 10 | 54 | 13 |

Notes: Each figure for acute, chronic and inpatient care represents the number of cases (3573, 2280 and 437 respectively) reported. The sample for chronic illnesses and inpatient care exclude children younger than 13 years of age. Responses are not mutually exclusive.
