# Supplementary material for: Healthcare seeking behaviour among self-help group households in Rural Bihar and Uttar Pradesh, India
Source: BMC Health Serv Res. 2016 Jan 4;16:1. doi: 10.1186/s12913-015-1254-9 (PMC4698810; doi:10.1186/s12913-015-1254-9)
Supplement: Supplementary file 3 — Predicted means/standard deviations (SD) of estimated travel time and cost by provider. (DOCX 14 kb) [file 12913_2015_1254_MOESM3_ESM.docx]

## Supplementary Table 3: Predicted means/standard deviations (SD) of estimated travel time and cost by provider

| Variable | Other | | NDAP | | Pharmacist | | Public | | Private | |
| --- | --- | --- | --- | --- | --- | --- | --- | --- | --- | --- |
|  | Mean | SD | Mean | SD | Mean | SD | Mean | SD | Mean | SD |
| Pooled Data |  |  |  |  |  |  |  |  |  |  |
| Average travel time to the provider (minutes) | 24 | 32 | 17 | 20 | 19 | 19 | 39 | 54 | 38 | 41 |
| Average cost for first visit (in INR) for acute illness | 81 | 124 | 128 | 210 | 69 | 189 | 155 | 301 | 380 | 1117 |
| Average cost for first visit (in INR) for chronic illness | 304 | 780 | 246 | 378 | 154 | 278 | 570 | 1130 | 929 | 1743 |
| Kanpur Dehat |  |  |  |  |  |  |  |  |  |  |
| Average travel time to the provider (minutes) | 17 | 31 | 24 | 26 | 29 | 30 | 56 | 45 | 54 | 51 |
| Average cost for first visit (in INR) for acute illness | 85 | 130 | 148 | 251 | 83 | 174 | 213 | 480 | 454 | 1832 |
| Average cost for first visit (in INR) for chronic illness | 420 | 1211 | 312 | 490 | 103 | 139 | 645 | 1055 | 1245 | 2741 |
| Pratapgarh |  |  |  |  |  |  |  |  |  |  |
| Average travel time to the provider (minutes) | 26 | 23 | 18 | 15 | 18 | 12 | 34 | 61 | 32 | 34 |
| Average cost for first visit (in INR) for acute illness | 108 | 150 | 105 | 139 | 46 | 62 | 123 | 166 | 208 | 300 |
| Average cost for first visit (in INR) for chronic illness | 153 | 171 | 224 | 335 | 139 | 285 | 376 | 1020 | 671 | 1248 |
| Vaishali |  |  |  |  |  |  |  |  |  |  |
| Average travel time to the provider (minutes) | 28 | 37 | 9 | 12 | 16 | 18 | 32 | 39 | 32 | 34 |
| Average cost for first visit (in INR) for acute illness | 64 | 105 | 129 | 219 | 92 | 275 | 176 | 324 | 434 | 774 |
| Average cost for first visit (in INR) for chronic illness | 449 | 773 | 215 | 297 | 209 | 287 | 902 | 1331 | 970 | 1325 |
